# Selenium suppressed the LPS‐induced inflammation of bovine endometrial epithelial cells through NF‐κB and MAPK pathways under high cortisol background

**DOI:** 10.1111/jcmm.17738

**Published:** 2023-04-11

**Authors:** Luying Cui, Jiaqi Zhang, Jing Guo, Min Zhang, Wenjie Li, Junsheng Dong, Kangjun Liu, Long Guo, Jun Li, Heng Wang, Jianji Li

**Affiliations:** ^1^ College of Veterinary Medicine, Yangzhou University Jiangsu Co‐Innovation Center for Prevention and Control of Important Animal Infectious Disease and Zoonoses Yangzhou 225009 China; ^2^ Joint International Research Laboratory of Agriculture and Agriproduct Safety of the Ministry of Education Yangzhou Jiangsu 225009 China; ^3^ International Research Laboratory of Prevention and Control of Important Animal Infectious Diseases and Zoonotic Diseases of Jiangsu Higher Education Institutions Yangzhou University Yangzhou 225009 China

**Keywords:** bovine endometrial epithelial cells, cortisol, inflammation, MAPK, NFκB, selenium

## Abstract

The bovine uterus is susceptible to infection, and the elevated cortisol level due to stress are common in cows after delivery. The essential trace element selenium plays a pivotal role in the antioxidant and anti‐inflammatory defence system of body. This study investigated whether selenium supplementation protected endometrial cells from inflammation in the presence of high‐level cortisol. The primary bovine endometrial epithelial cells were subjected to *Escherichia coli* lipopolysaccharide to establish cellular inflammation model. The gene expression of inflammatory mediators and proinflammatory cytokines was measured by quantitative PCR. The key proteins of NF‐κB and MAPK signalling pathways were detected by Western blot and immunofluorescence. The result showed that pre‐treatment of Na_2_SeO_3_ (1, 2 and 4 μΜ) decreased the mRNA expression of proinflammatory genes, inhibited the activation of NF‐κB and suppressed the phosphorylation of extracellular signal‐regulated kinase, P38MAPK and c‐Jun N‐terminal kinase. This inhibition of inflammation was more apparent in the presence of high‐level cortisol (30 ng/mL). These results indicated that selenium has an anti‐inflammatory effect, which is mediated via NF‐κB and MAPK signalling pathways and is augmented by cortisol in bovine endometrial epithelial cells.

## 1 INTRODUCTION

Uterine health is often compromised in cattle because postpartum contamination of the uterine lumen by bacteria is ubiquitous, and pathogenic bacteria frequently persist causing clinical diseases.^1^ Postpartum metritis and endometritis are important disorders in cattle,^2^, ^3^ causing subfertility and infertility^4^ and high economic losses due to the lower oestrus expression and prolonged calving intervals, resulting in involuntary culling.^5^

The presence of pathogenic bacteria in the uterus causes inflammation and histological lesions of the endometrium, delays uterine involution and perturbs embryo survival.^6^ The recognized uterine pathogens most often associated with clinical disease are *Arcanobacterium pyogenes*, *Escherichia coli* (*E. coli*), *Fusobacterium necrophorum* and *Prevotella melaninogenicus*.^1^ Thus, *Arcanobacterium pyogenes* and *E. coli* are often used to induce uterine infections.^7^ There is general agreement that lipopolysaccharide (LPS), a cell wall component of Gram‐negative bacteria, is the main pathogenic factor involved in *E. coli* infection.^8^ Recognition of LPS by the innate immune system is critical to elicit inflammatory responses.^9^ Host cells recognize LPS via a specific receptor complex comprising of Toll‐like receptor 4 (TLR4), CD14 and MD‐2, which leads to an inflammatory response characterized with the secretion of cytokines and chemokines.^10^ Endometrial epithelial and stromal cells express the TLR4 complex. LPS stimulates the secretion of chemokines such as interleukin (IL)‐8 and disrupts endocrine function by switching prostaglandin secretion to predominantly prostaglandin E_2._ ^11^, ^12^ TLR4 recognizes LPS and recruits adaptor protein MyD88 to initiate the MyD88‐dependent pathway, which involves a series of signal transduction intermediators and activates the nuclear factor‐κB (NF‐κB) to induce the production of inflammatory cytokines such as tumour necrosis factor (TNF)‐α, IL1β and IL6. Meanwhile, LPS is also a potent activator of the three classical mitogen‐activated protein kinase (MAPK) pathways, including extracellular signal‐regulated kinase (ERK1/2), P38MAPK and c‐Jun N‐terminal kinase (JNK).^13^ These signalling pathways in turn promote transcription of genes encoding *TNF*, *IL1B*, *IL6*, C‐X‐C motif chemokine ligand 8 (*CXCL8*), inducible nitric oxide synthase 2 (*NOS2*) and prostaglandin‐endoperoxide synthase 2(*PTGS2*) and finally causing inflammation.^14^, ^15^ In bovine endometrial epithelial cells (BEEC), TLR4 and MyD88‐dependent signalling pathway are critical to the response of LPS.^16^

Selenium (Se) is an essential micronutrient for animals and human and plays a critical role in the anti‐inflammatory process and the antioxidant defence system.^17^, ^18^ Studies have demonstrated an enhancement of both cell‐mediated and humoral immune responses by increasing Se intake and that Se supplementation promoted the activities of natural‐killer cells and cytotoxic T‐cells.^19^, ^20^ Se supplementation have been observed in vitro to suppress NF‐κB activation and the downstream proinflammatory gene expression following ultraviolet treatment and promote the apoptosis in tumour cells, the phytohemagglutinin response in lymphocytes, the function of phagocytosis in macrophages and the killing capacity of cytotoxic T‐cells.^20^ Se inhibited NF‐κB activation and the subsequent inflammation through modulating selenoprotein production in human T lineage cells and human hepatoma cell line HuH‐7.^19^, ^21^ Se supplementation resulted in a significant decrease in LPS‐induced productions of TNF, reactive oxidative species (ROS) and cyclooxygenase‐2 (COX‐2) by inhibiting MAPK signalling.^22^, ^23^, ^24^ However, whether Se supplementation could inhibit the activation of NF‐κB and MAPK signalling pathway induced by LPS in BEEC has not been reported.

Serum cortisol concentrations are higher in postpartum cows due to stress.^25^ Cortisol is well‐recognized as an anti‐inflammation agent.^26^ A mild increase of cortisol level may serve to minimize the inflammatory tissue damage and encourage the rapid healing of the ovarian surface to anticipation of the next ovulatory cycle.^27^ However, exposure to high‐level cortisol exacerbates the state of immunosuppression.^28^ Previous studies found that cortisol inhibited the activation of NF‐κB and MAPK signalling, and the expression of inflammatory mediators and proinflammatory cytokines in LPS‐induced RAW264.7 macrophages and BEEC.^29^ However, neither the anti‐inflammatory activity of Se on BEEC at high cortisol background nor the mechanism of this process has been reported.

The aim of this study was to investigate the influence of Se on the inflammatory response of primary BEEC with high cortisol background. Here, we observed that Na_2_SeO_3_ relieved the inflammation of BEEC through NF‐κB and MAPK pathways, and this anti‐inflammatory effect of Se was augmented by the presence of cortisol.

## 2 MATERIALS AND METHODS

### 2.1 Culture of primary bovine endometrial epithelial cells

All operations comply with animal welfare regulations. The primary BEEC were isolated as described from previous reports.^29^, ^30^ Bovine uteri of non‐pregnant cows free of genital diseases or microbial infections were collected from local abattoir. After collection, the uteri surfaces were rinsed with iodophor and stored frozen until the further step in the laboratory. Briefly, the uterine horns were cut into 3–4 cm strips on a sterile operating table, and the uterine tissue was digested and diluted in DMEM/F12 (D8900, Sigma, USA) with 0.1% protease from *Streptomyces griseus* (P5147, Sigma, USA). After digestion at 4°C for 18 h, the uterine tissues were removed and placed on aseptic culture dishes, and the endometrium was washed in phosphate‐buffered saline (pH values from 7.2 to 7.4) by scraping with a sterile scalpel blade. Then the washed suspension was collected and centrifuged at 290*g* for 5 min, and the supernatant was discarded. This step was repeated 3 times until the supernatant was not cloudy. The cells were cultured in DMEM‐F12 medium containing 15% foetal bovine serum (FBS, Gibco), 50 U/mL penicillin/streptomycin and seeded into a 25 cm^2^ flasks then incubated at 37°C, 5% CO_2_ and 95% sterile air. The medium was changed every 1–2 days until the cells reached approximately 90% confluence.

### 2.2 Experiment design and treatments

The sodium selenite (Na_2_SeO_3_) used in this study is a lyophilized powder synthesized by Sigma (S5261). The powder was dissolved in DMEM/F‐12, filtered and diluted to a concentration of 64 μM and was stored at −20°C. Selection of the concentration of Na_2_SeO_3_ in the range of 1–64 μM was based on previous studies.^31^ The physiological level of cortisol concentration in cattle ranges from 5 to 30 ng/mL. Based on a preliminary study in our laboratory,^29^ a concentration of 30 ng/mL was selected as the high cortisol level.

**FIGURE 1:**
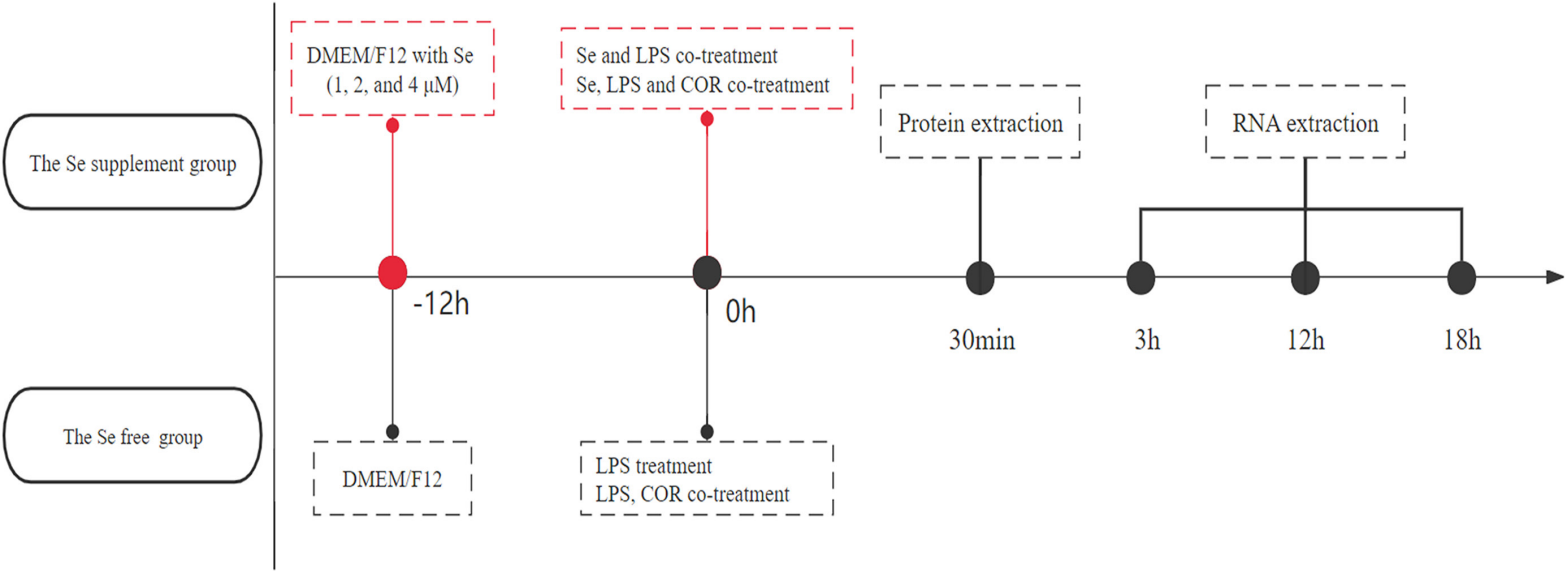
Study timeline illustrating the time point of selenium (Se), lipopolysaccharide (LPS) and/or cortisol (COR) treatment. The primary bovine endometrial epithelial cells were collected at 0.5, 3, 12 and 18 h after LPS stimulation.

The experiment 1 explored the effect of Se on NF‐κB and MAPK pathways and the downstream cytokine expression. First, the cells of experimental groups were pre‐treated with medium containing 1, 2 and 4 μM Na_2_SeO_3_ for 12 h.^31^ Cell viability and growth rates of Se‐supplemented cells were similar to their counterparts treated with control medium alone. Then the cells were treated with LPS (1 μg/mL)^15^ or co‐treated with Na_2_SeO_3_. The group were as follows: the blank control group, the LPS treatment group, the Se (4 μM) treatment group, the LPS and Se (1, 2 and 4 μM) co‐treatment group. In experiment 2, we observed the effect of Se on the inflammatory response in LPS‐stimulated BEEC under high cortisol background. After Se pre‐treatment for 12 h, the cells were challenged with LPS (1 μg/mL) and cortisol (30 ng/mL). The group were as follows: the blank control group, the LPS group, the LPS and cortisol co‐treatment group, the LPS, cortisol and Se (1, 2 and 4 μM) co‐treatment groups. The experimental design was shown in Figure 1.

### 2.3 Cell viability assay

The effect of Na_2_SeO_3_ on BEEC viability was measured by Cell Counting Kit‐8 (CCK‐8, Dojindo Molecular Technologies, Inc.). The cells were plated into 96‐well plates at a density of 1 × 10^3^ cells per well and grown to 80% confluence in a 37°C, 5% CO_2_ incubator. Then the cells of Se supplement groups were treated with DMEM/F12, which containing various concentrations of Na_2_SeO_3_ (1, 2, 4, 8, 16, 32 and 64 μM) for 12 h. After treatment, CCK‐8 was added to each well, and the cells were incubated at 37°C for 2 h. The optical density was read at 450 nm using a microplate reader (Tecan, Austria).

The lactate dehydrogenase (LDH) in cell culture medium was detected using a LDH assay kit (Jiancheng Bioengineering Research Institute) to determine cytotoxicity of Na_2_SeO_3_. The cells were seeded into 6‐well plates at a density of 1 × 10^6^ cells per well and grown to 80% confluent at 37°C with 5% CO_2_. Then the cells of Se supplement groups were treated with DMEM/F12 containing Na_2_SeO_3_ (1–64 μM) for 12 h. Then the cell culture medium was collected. According to the instructions of the kit, the absorbance value was read at the wavelength of 450 nm with a microplate reader.

### 2.4 RNA extraction and quantitative PCR

The BEEC was seed in 6‐well plates (1 × 10^6^ cells/well), and the cells were grown to 80% confluence. The BEEC was collected at 3, 12 and 18 h posttreatment^30^ and the RNA was extracted using TRIzol reagent (R401‐01, Vazyme, China). The extracted RNA was quantified using a Nanodrop 2000 spectrophotometer (Thermo, USA), and the A260/280 ratio of each sample was between 1.8 and 2.1. The RNA was converted to cDNA using a reverse transcriptase synthesis kit (DRR047A, TaKaRa, Japan). QPCR was performed using a CFX 96 Real‐Time PCR Detection System (BIO‐RAD, USA). Amplification mixtures contained 10 μL of 2× ChamQ SYBR qPCR Master mix (Q311‐02/03, Vazyme, China), 4 μL of each primer and 2 μL of cDNA template in a final volume of 20 μL per reaction (RR820A, TaKaRa, Japan). The sequences of the primers were presented in Table 1. The expression of each gene was normalized against the housekeeping gene *ACTB*. The 2−^△△CT^ method was used to calculate the relative expression of the genes.^32^

**TABLE 1:** The list of primer sequences

Target gene	Primer sequences (5′ ‐3′)	Accession number
*ACTB*	F: CATCACCATCGGCAATGAGC	NM_173979.3
	R: AGCACCGTGTTGGCGTAGAG	
*IL1B*	F: AGGTCCATACCTGACGGCTA	NM_174092.1
	R: TTGGGTGTCTCAGGCATCTC	
*IL6*	F: TGAAAGCAGCAAGGAGACACT	NM_173923.2
	R: TGATTGAACCCAGATTGGAAGC	
*CXCL8*	F: TTCCTCAGTAAAGATGCCAATG	NM_173925.2
	R: TGACAACCCTACACCAGACCCA	
*TNF*	F: CCACGTTGTAGCCGACATC	NM_173966.3
	R: CCCTGAAGAGGACCTGTGAG	
*NOS2*	F: ACCTACCAGCTGACGGGAGAT	NM_001076799.1
	R: TGGCAGGGTCCCCTCTGATG	
*PTGS2*	F: CCAGAGCTCTTCCTCCTGTG	NM_174445.2
	R: AAGCTGGTCCTCGTTCAAAA	

### 2.5 Western blot analysis

The protein extraction time point was based on previous reports.^29^, ^30^ The protein concentration was determined using the bicinchoninic acid protein assay kit (P0010, Beyotime). The protein (20–30 μg) was separated on 9% SDS‐polyacrylamide gels and transferred to polyvinylidene difluoride membranes (Millipore, Germany). The membranes were incubated with tris‐buffered saline containing 0.05% Tween 20% and 5% non‐fat milk to block the nonspecific binding. Antibodies specific for p‐P65 (# 3033), P65 (# 8242), p‐IκBα (# 2859), IκBα (# 4812), p‐ERK1/2 (# 4370), ERK1/ 2 (# 4695), p‐P38 (# 4511), P38 (# 8690), p‐JNK (# 4668), JNK (# 9258), GAPDH (#8884) and β‐actin (# 4970) were purchased from Cell Signalling Technology (Danvers, MA, USA). The membranes were incubated with primary antibodies, which were all diluted at 1:1000 with antibody diluent (Abs954, Absin, China) at 4°C overnight, followed by the incubation with the HRP‐conjugated goat anti‐rabbit secondary antibody (#7074, Cell Signalling Technology) at 1:2000 dilution in 5% non‐fat milk for 1 h at room temperature. The proteins were detected using a chemiluminescence assay. The antigen–antibody complexes were visualized on horseradish peroxidase substrate (Millipore) using the ChemiScope5300Pro CCD camera (Clinx Science Instruments). The band intensity was quantified by Quantity One software (Bio‐Rad).

### 2.6 Immunofluorescence staining

The BEEC was plated on cover glasses in 24‐well cell plates (1 × 10^4^ cells/well). After treatment, the cells were fixed with 4% paraformaldehyde (BL539A, Biosharp) for 15 min, followed by permeabilization with 0.1% Triton X‐100 (ST797，Beyotime，China) for 10 min. After blocking with 5% Albumin Bovine V (#A8020, Solarbio, China) for 1 h at room temperature, the cells were incubated at 4°C overnight with NF‐κB P65 (all at 1:250 in antibody diluent). The cover glasses were washed with phosphate‐buffered saline, and incubation with FITC‐conjugated secondary antibody (A0423, Beyotime, China) for 1 h at room temperature. Finally, the cells were dyed with DAPI and examined using a fluorescence confocal microscopy (Leica TCS SP8 STED, Leica Corporation, Germany). To determine P65 intensity in the nucleus, the immunofluorescence signals were quantified by Image J software (National Institutes of Health).

### 2.7 Statistical analysis

Each experiment was repeated three times. All data were analysed using IBM SPSS Statistics 21.0 (IBM) and were expressed as the means ± standard error of means (SEM). Statistically significant differences throughout this study were calculated by one‐way anova, followed by Dunnett's test. A two‐sided *p* value <0.05 was considered statistically significant.

## 3 RESULTS

### 3.1 Cell viability and LDH release

**FIGURE 2:**
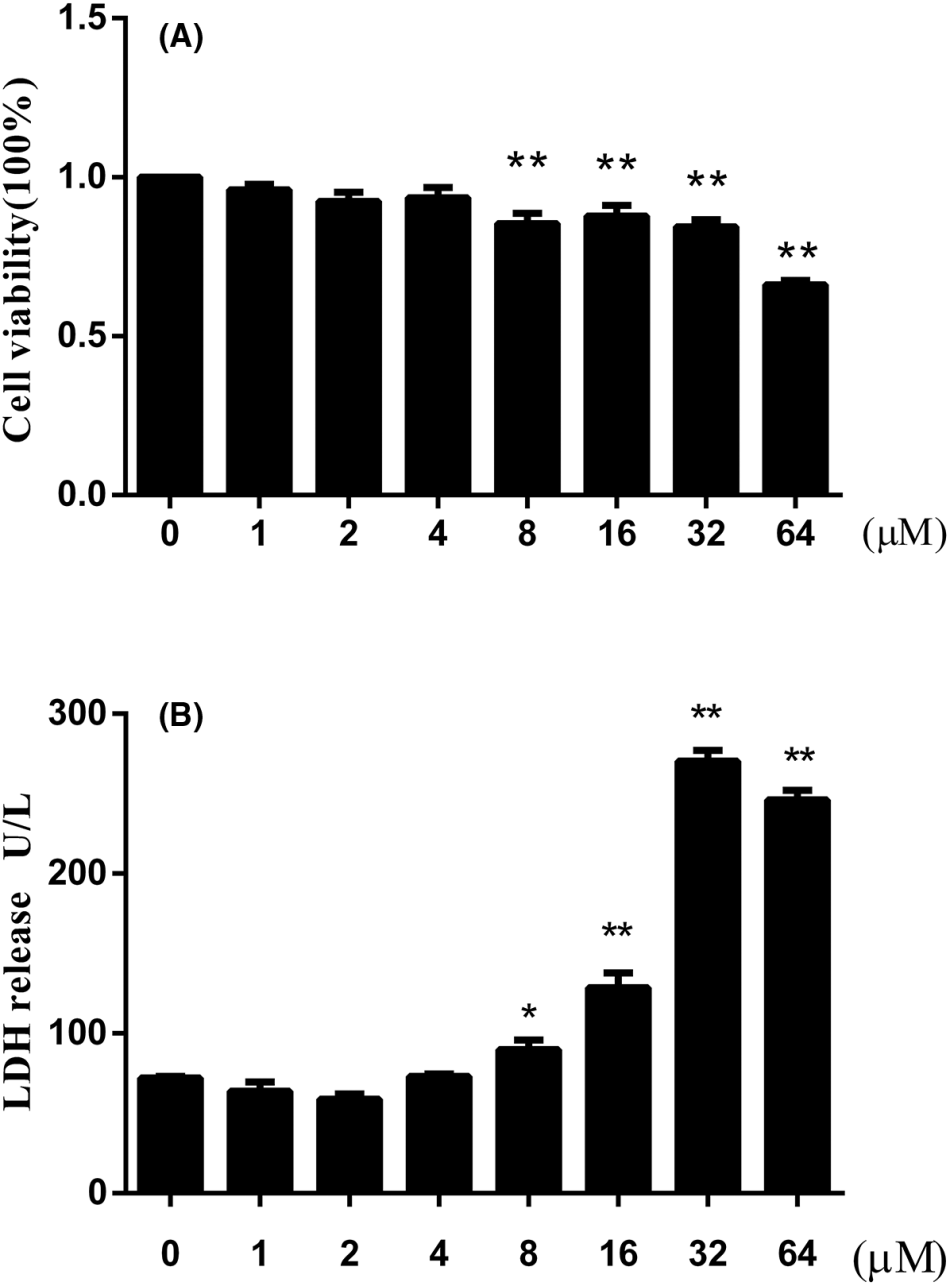
The effect of different concentrations of Na_2_SeO_3_ (1–64 μM) on cell viability (A) and LDH release (B) of primary bovine endometrial epithelial cells. Data were presented as means ± SEM (*n* = 6). LDH, lactate dehydrogenase. *p < 0.05, **p < 0.01 versus the blank group.

**FIGURE 3:**
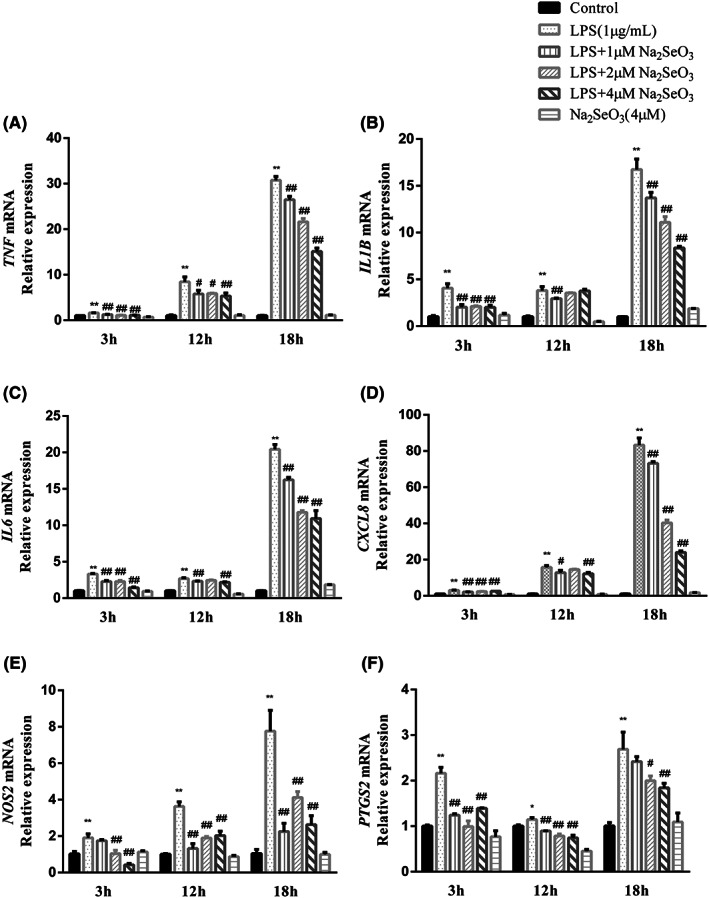
Variations in mRNA expression of *NOS2*, *PTGS2* and inflammatory cytokines in primary bovine endometrial epithelial cells (A–F) co‐treated with 1 μg/mL LPS and 1, 2, 4 μM Na_2_SeO_3_ for 3, 12 and 18 h. Data were presented as means ± SEM (*n* = 3). CXCL8, C‐X‐C motif chemokine ligand 8; IL, interleukin; *NOS2*, inducible nitric oxide synthase 2; *PTGS2*, prostaglandin‐endoperoxide synthase 2; TNF, tumour necrosis factor. *p < 0.05, **p < 0.01 versus the control group. #*p* < 0.05, ##*p* < 0.01 versus the LPS group.

The effect of different concentrations of Na_2_SeO_3_ on cell viability and LDH release was shown in Figure 2. Compared to the blank control, the LDH release increased (*p* < 0.05) and cell viability decreased (*p* < 0.01) when the Se concentration exceeded 4 μM. The cell viability was around 85% in 8 μM Se group and only about 66% in 64 μM Se group (Figure 3A). Furthermore, no difference was found between the control group and the group treated with 1, 2 and 4 μM Na_2_SeO_3_.

### 3.2 Gene expression of *NOS2* , *PTGS2* and proinflammatory cytokines

To determine the effect of Se on the inflammatory response of BEEC induced by LPS, we examined the mRNA expression of *TNF*, *IL‐1B*, *IL6*, *CXCL8*, *NOS2* and *PTGS2*. As depicted in Figure 3, the expression of inflammatory cytokines and chemokines of the LPS group was upregulated (*p* < 0.01) compared with the control group at 3, 12 and 18 h, and reached peak at 18 h, with 5–20 times higher than those of the control group. Meanwhile, compared with the LPS group, the mRNA expression of these genes was downregulated (*p* < 0.05) by Se supplement at the indicated time points. In addition, among the three Se concentrations, 4 μM Se seemed to show the most prominent inhibitory effect on these proinflammatory genes. Compared the control group, none (*p* > 0.05) of the gene expression was affected by Se alone.

**FIGURE 4:**
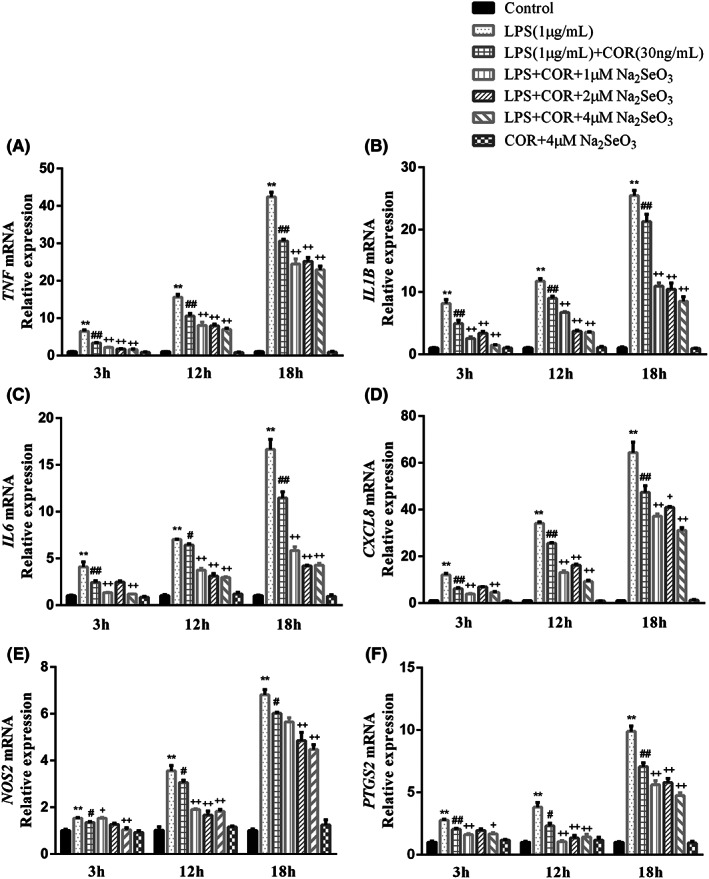
Variations in mRNA of *NOS2*, *PTGS2* and inflammatory cytokines in bovine endometrial epithelial cells (BEEC, A–F) co‐treated with 1 μg/mL LPS, 30 ng/mL cortisol (COR) and 1, 2, 4 μM Na_2_SeO_3_ for 3, 12, 18 h. Data were presented as means ± SEM (*n* = 3). CXCL8, C‐X‐C motif chemokine ligand 8; IL, interleukin; *NOS2*, inducible nitric oxide synthase 2; *PTGS2*, prostaglandin‐endoperoxide synthase 2; TNF, tumour necrosis factor. *p < 0.05, **p < 0.01 versus the control group. #*p* < 0.05, ##*p* < 0.01 versus the LPS group. +*p* < 0.05, ++*p* < 0.01 versus the LPS‐COR group.

The influence of Se on the inflammatory response of BEEC under high cortisol level was shown in Figure 4. Similar to the previous result, LPS stimulation resulted in a marked increase (*p* < 0.01) of these gene expression. Cortisol of 30 ng/mL downregulated (*p* < 0.05) all these gene expression. Compared with the LPS and cortisol co‐treatment group, co‐incubation with LPS, cortisol and different concentrations of Se further reduced (*p* < 0.05) these gene expressions except (*p* > 0.05) the *IL6*, *CXCL8*, *NOS2* and *PTGS2* mRNA in cells supplemented with 2 μM Na_2_SeO_3_ at 3 h. What's more, 4 μM Se showed the best inhibitory effect among the three Se supplement groups. Co‐treatment with cortisol and 4 μM Se did not cause any change (*p* > 0.05) in the mRNA expression of these proinflammatory factors compared with the control group.

### 3.3 NF‐κB activation and MAPK phosphorylation

**FIGURE 5:**
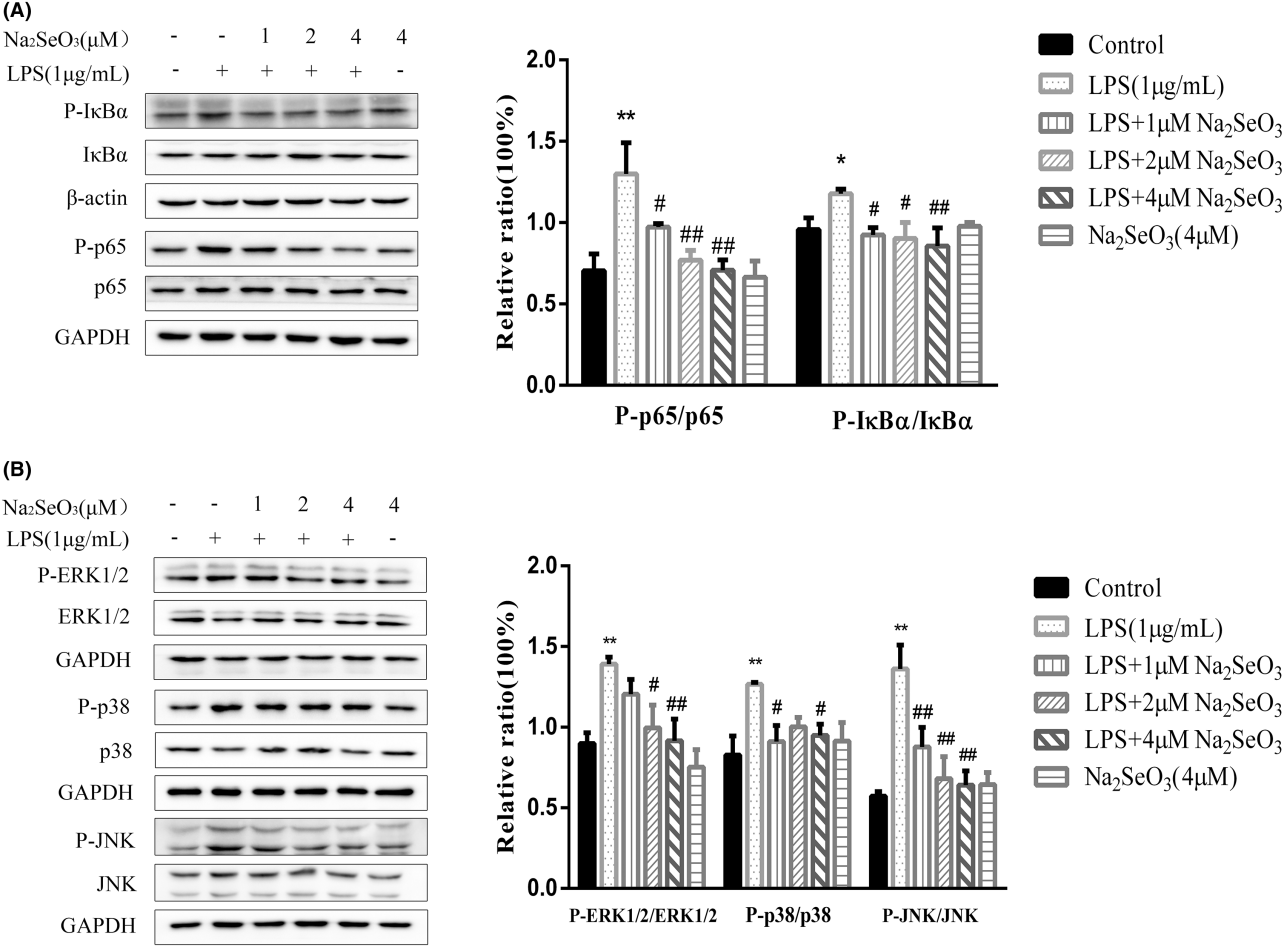
The effect of selenium on LPS‐stimulated NF‐κB activation (A) and MAPK phosphorylation (B) in primary bovine endometrial epithelial cells co‐treated with 1 μg/mL LPS and 1, 2, 4 μM Na_2_SeO_3_ for 30 min. Data were presented as means ± SEM (*n* = 3). *p < 0.05, **p < 0.01 versus the control group; #*p* < 0.05, ##*p* < 0.01 versus the LPS group.

To investigate the mechanism of Se on the inflammatory status of BEEC, we determined the critical proteins of NF‐κB and MAPK signalling pathways by Western blotting. As shown in Figures 4, 5 μM Se alone caused no change (*p* > 0.05) in the phosphorylation level of key protein of NF‐κB and MAPK pathway in BEEC. LPS challenge increased (*p* < 0.05) the ratios of p‐P65/P65 and p‐IκBα/IκBα and promoted (*p* < 0.01) the phosphorylation of ERK1/2, P38 and JNK in BEEC. Se supplementation generally inhibited (*p* < 0.05) the activation of NF‐κB and MAPK, and 4 μM Se reduced the phosphorylation level of key proteins to 0.5–0.7 fold.

**FIGURE 6:**
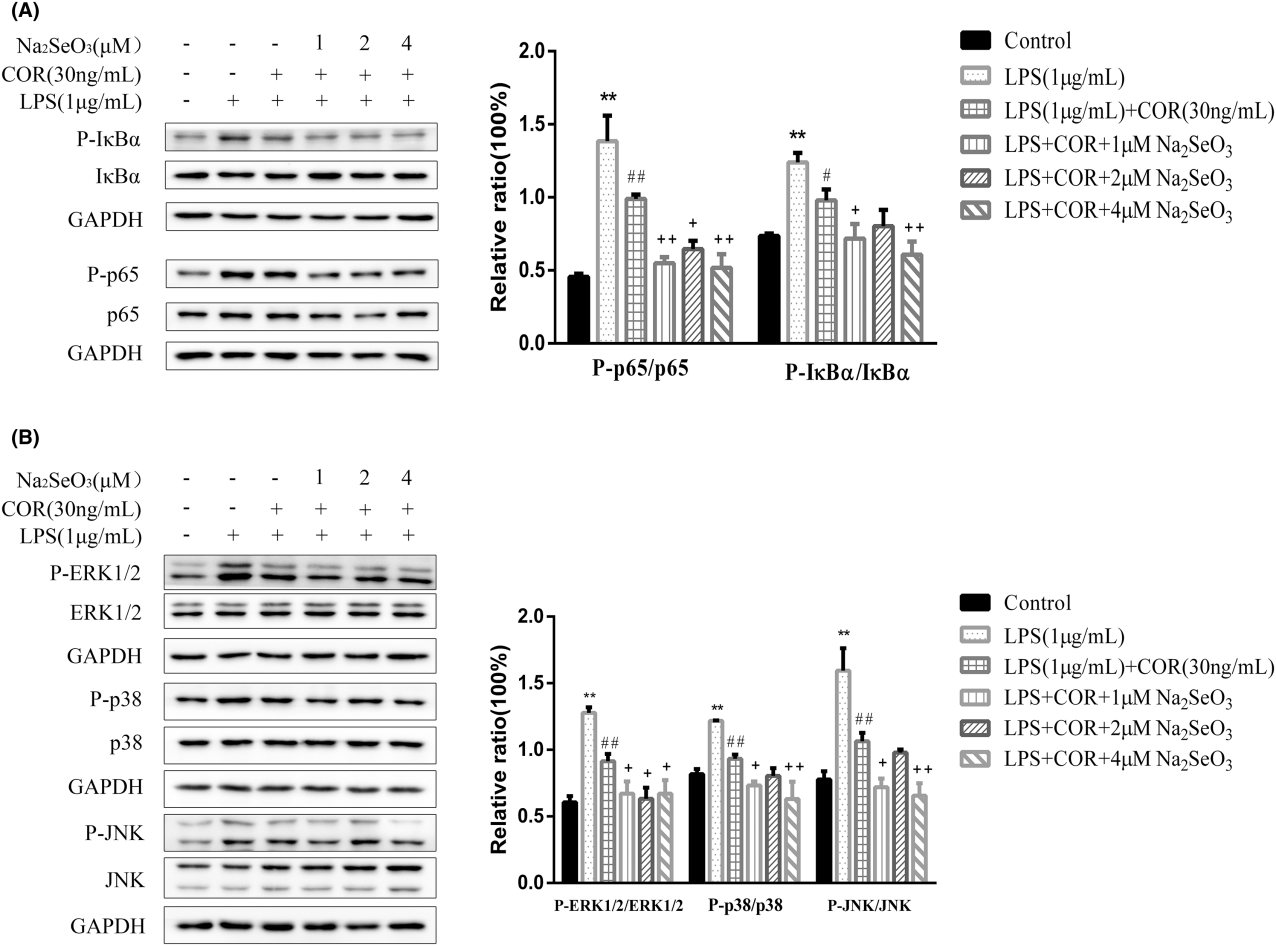
The effect of selenium on LPS‐stimulated NF‐κB activation (A) and MAPK phosphorylation (B) in primary bovine endometrial epithelial cells in high cortisol background. The cells were co‐treated with 1 μg/mL LPS, 30 ng/mL cortisol (COR) and Na_2_SeO_3_ of 1, 2 or 4 μM for 30 min. Data were presented as means ± SEM (*n* = 3). *p < 0.05, **p < 0.01 versus the control group; #*p* < 0.05, ##*p* < 0.01 versus the LPS group; +*p* < 0.05, ++*p* < 0.01 versus the LPS‐COR group.

As shown in Figure 6, compared with the LPS and cortisol co‐treatment group, co‐incubation with LPS, cortisol, and various concentrations of Se further reduced (*p* < 0.05) the phosphorylation of these critical proteins. The co‐treatment group with a Se concentration of 4 μM reduced the level of LPS‐induced protein phosphorylation to 0.4–0.6 fold.

**FIGURE 7:**
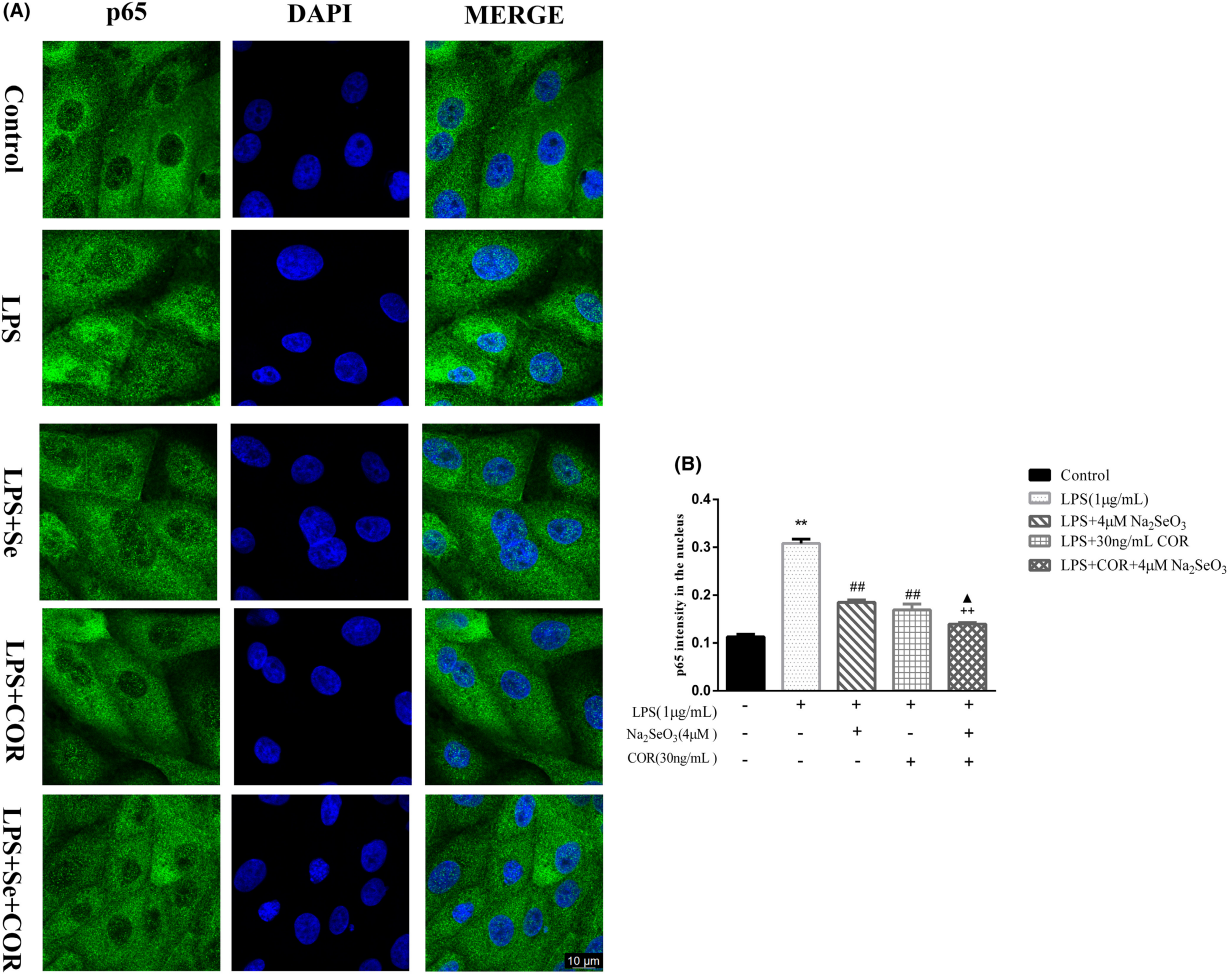
The effect of selenium or selenium and cortisol on the P65 translocation of primary bovine endometrial epithelial cells. The cells were co‐treated with 1 μg/mL LPS, 30 ng/mL cortisol (COR) and 4 μM selenium for 30 min. (A) The translocation of P65 from the cytoplasm into the nucleus was visualized by confocal microscopy; (B) Quantification above background levels of P65 intensity in the nucleus was analysed by Image J software. Scale bar represented 10 μm. Data were presented as means ± SEM (*n* = 3). **p < 0.01 versus the control group; ##*p* < 0.01 versus the LPS group; ++*p* < 0.01 versus the LPS‐Se group; ▲*p* < 0.05 versus the LPS‐COR group.

The immunofluorescence result was demonstrated in Figure 7. LPS promoted (*p* < 0.01) the nuclear translocation of P65 in BEEC. The supplementation of 4 μM Se or 30 ng/mL cortisol showed lower (*p* < 0.01) level of P65 in the nucleus in comparison with the LPS group. The level of P65 in the nucleus were the lowest in the co‐treatment group of LPS, Se, and cortisol compared to the group of LPS and Se (*p* < 0.01) or LPS and COR (*p* < 0.05).

## 4 DISCUSSION

The previous study of our lab found that Se alleviated the inflammatory reaction by decreasing TNF, IL1B and IL6 expression and inhibiting NF‐κB and MAPK activation in RAW264.7 macrophages stimulated with *Staphylococcus aureus*.^31^ In this study, we found that supplementation of Se downregulated the mRNA level of inflammatory mediators (*NOS2*, *PTGS2*) and inflammatory cytokines (*TNF*, *IL1B*, *IL6* and *CXCL8*) and inhibited the activation of NF‐κB and the phosphorylation of MAPK in BEEC challenged with LPS. Cortisol has been shown to exhibit anti‐inflammatory effects by suppressing NF‐κB and MAPK signalling pathways in BEEC.^29^ Here our results revealed that Se supplementation with high cortisol background attenuated LPS‐induced proinflammatory gene expression and had a stronger anti‐inflammatory effect than cortisol alone, and this inhibitive effect was possibly through the NF‐κB and MAPK signalling pathways, while the mechanisms of inhibition by Se and cortisol are likely to be different.

LPS is the major virulence factor of pathogenic Gram‐negative bacteria, including *E. coli*.^33^, ^34^ TLR4 has been identified as the pattern recognition receptor for *E. coli* LPS.^35^ Previous research has shown that TLR4 recognizes LPS to initiate the MyD88‐dependent pathway, which includes a series of signal transduction intermediators and activates the NF‐κB and MAPK to induce the production of inflammatory cytokines and these pathways have been found to synergize through synchronized binding to κB and AP‐1 sites found together in the promoters of many genes, including CXCL8.^15^, ^16^, ^36^ In our study, LPS stimulated the activation of NF‐κB and increased the phosphorylation level of key proteins of MAPK pathway and promoted the expression of inflammatory mediators and inflammatory cytokines.

Se is an essential trace mineral of fundamental importance for animal,^37^ and is known primarily for its antioxidant activity and in therapeutic aspects for its chemopreventive, anti‐inflammatory and antiviral properties.^38^ Se can be found as selenomethionine, selenocysteine, selenite, selenate and elemental selenium in human body. Se enters the human or animal body through the following chain: soil–plant–food. Hence, Se concentration in the human blood and tissues depends on the Se content in food. The average content of Se in plants is within 0.01–10 mg/kg dry mass and its amount is affected by the type of soil and its pH value, amount of precipitation, temperature and plant development phase. According to different geographical regions, the concentration of selenium in human serum varies in different regions of the world, including 5–7800 μg/L in China, 100–350 μg/L in USA, and 89–98 μg/L in Germany.^39^, ^40^ In order to identify whether Se supplementation has an influence on inflammation of BEEC, we used LPS to induce an inflammatory response. As a result, Se supplementation alleviated the inflammation by decreasing the gene expression of inflammatory mediators (*PTGS2*, *NOS2*) and proinflammatory cytokines (*IL1B*, *IL6*, *CXCL8*, *TNF*). Similarly, Se supplementation has been observed to downregulate COX‐2 at mRNA and protein level and the enzyme activity to exert its anti‐inflammatory effect in macrophages.^24^, ^41^, ^42^ Moein Ala et al. found that sodium selenite inhibited TLR4 and NF‐κB signalling and the inflammatory cytokine (TNF‐α, INF‐γ, IL17 and IL22) release in rats with acetic acid‐induced colitis.^43^

The release of cortisol is a primary adaptive mechanism in response to adverse conditions through the short‐term mobilization of body reserves and modulation of inflammatory responses.^44^ Parturition and dystocia causes elevated cortisol release.^45^ In addition, the decreased food intake from 1–2 weeks before calving also reduces the availability of antioxidants in the body. Antioxidant supplementation during the peripartum period is especially important for cattle,^46^ and the improvement of antioxidative status could be one important mechanism supporting the immune function of dairy cows during peripartum period.^47^ Therefore, we investigated the effect of Se supplementation on the inflammatory response of BEEC in a high‐level cortisol state. In agreement with previous reports, cortisol suppressed the inflammatory response in BEEC.^29^ More importantly, we observed that Se supplement further decreased the proinflammatory gene expression and inhibited the NF‐κB and MAPK activation. This coincided with the report that supplementation of antioxidants such as vitamin E and Se during pregnancy relieved the inflammation in dystocia‐affected buffaloes during the immediate postpartum period.^48^ In the form of selenoprotein, Se has been confirmed to inhibit inflammation through NF‐κB and MAPK pathways.^49^, ^50^ By suppressing IκBα phosphorylation and lengthening the half life of IκBα, Se inhibited the translocation of P65 NF‐κB subunit to the nucleus,^51^ thereby impeding the transcription of proinflammatory genes. The direct interaction between Se and MAPK signalling was less reported. MAPK is considered to be an important downstream signalling pathway of ROS.^52^ Given that Se acts as an antioxidant through selenoproteins and reduces cellular oxidative damage,^53^ the inhibitive effect of Se on MAPK signalling is probably achieved by ROS elimination in BEEC. Recent studies in our lab have shown that cortisol reduced the production of ROS (data unpublished, Supplementary file 1), which further supports this view.

Cortisol has been verified to suppress LPS‐induced inflammation in BEEC by inhibiting NF‐κB and MAPK signalling pathways.^29^ In addition, the anti‐inflammatory effect of 4 μM Se was more pronounced in the high cortisol state, revealing a synergistic effect of Se and cortisol to alleviate the inflammation. These results indicated that selenium and cortisol exert anti‐inflammatory effects through NF‐κB and MAPK signalling pathway in BEEC and that the mechanism of inflammation inhibition by Se may be different from that of cortisol in BEEC.

Interestingly, in this study, we found that 2 μM Se had a less pronounced effect on expression levels of inflammatory cytokines (*IL1B*, *IL6*, *CXCL8* and *NOS2*) and phosphorylation levels of IκBα, P38 and JNK proteins compared to 1 μM and 4 μM Se, which was not in line with our expectations. There are no experiments showing a dose‐dependent response of Se supplementation to LPS‐induced inflammation in BEEC. Observations to date suggest that oxidative stress is closely linked to inflammation and modulates inflammation at its different development stages.^54^ Through its incorporation into selenoproteins, Se is involved in regulating ROS and redox status in nearly all immune responses,^17^ with effects on inflammatory pathways that are directly or indirectly influenced by its antioxidant mechanisms. According to the Se concentration, there are variations in the antioxidant capacity, and the level and range of the effect on inflammatory pathways. However, specifically for BEEC, the anti‐inflammatory and antioxidant link requires further investigation and should be testified in vivo. The question why the 2 μM Se treatment group had such effect in BEEC cannot be answered with the present dataset. Due to the complex interaction between selenium and cortisol, our results may point toward several future research directions, including the concentration‐specific effects of Se on selenoprotein expression and the determination of the exact mechanism of anti‐inflammation by selenium and cortisol through using pathway inhibitors and gene silencing techniques.

In summary, Se inhibited the LPS‐induced inflammatory response by suppressing NF‐κB and MAPK in BEEC, and this inhibitive property of Se was augmented with high cortisol background.

## AUTHOR CONTRIBUTIONS

**Luying Cui:** Conceptualization (equal); funding acquisition (equal); supervision (equal); writing – review and editing (equal). **Jiaqi Zhang:** Data curation (equal); formal analysis (equal); methodology (equal); writing – original draft (equal). **Jing Guo:** Data curation (equal). **Min Zhang:** Data curation (equal). **Wenjie Li:** Data curation (equal). **Junsheng Dong:** Funding acquisition (equal). **Kangjun Liu:** Methodology (equal). **Long Guo:** Methodology (equal). **Jun Li:** Supervision (equal). **Heng Wang:** Funding acquisition (equal). **Jianji Li:** Conceptualization (equal); funding acquisition (equal); supervision (equal).

## FUNDING INFORMATION

This work was supported by the National Natural Science Foundation of China (NO: 32072937, 31802253, 32102735); the China Postdoctoral Science Foundation (NO: 2018 M632398); the earmarked fund for Jiangsu Agricultural Industry Technology System (JATS[2022]499); 333 High‐level Talent Training Project of Jiangsu Province, China; the Natural Science Foundation of Jiangsu Province (NO: BK20210808); The Postgraduate Training Innovation Program of Jiangsu Province (KYCX21_3279); the 111 Project (D18007); and the Priority Academic Program Development of Jiangsu Higher Education Institutions (PAPD).

## CONFLICT OF INTEREST STATEMENT

The authors declare that there is no conflict of interest regarding the publication of this manuscript.

## Supporting information


Supinfo

## Data Availability

The data that support the findings of this study are available from the corresponding author upon reasonable request.

## References

[1] Sheldon IM , Dobson H . Postpartum uterine health in cattle. Anim Reprid Sci. 2004;82:295‐306. 10.1016/j.anireprosci.2004.04.006 15271461

[2] Melendez P , McHale J , Bartolome J , Archbald LF , Donovan GA . Uterine involution and fertility of Holstein cows subsequent to early postpartum PGF2alpha treatment for acute puerperal metritis. J Dairy Sci. 2004;87(10):3238‐3246. 15377603 10.3168/jds.S0022-0302(04)73460-8

[3] Földi J , Kulcsár M , Pécsi A , et al. Bacterial complications of postpartum uterine involution in cattle. Anim Reprod Sci. 2006;96(3–4):265‐281. 16956738 10.1016/j.anireprosci.2006.08.006

[4] Sheldon IM , Owens SE . Postpartum uterine infection and endometritis in dairy cattle. Anim Reprod. 2017;14(3):622‐629.

[5] Esslemont RJ , Peeler EJ . The scope for raising margins in dairy herds by improving fertility and health. Br Vet J. 1993;149(6):537‐547. 8111614 10.1016/S0007-1935(05)80038-7

[6] Sheldon IM , Lewis GS , Leblanc S , et al. Defining postpartum uterine disease in cattle. Theriogenology. 2006;65(8):1516‐1530. 16226305 10.1016/j.theriogenology.2005.08.021

[7] Lewis GS . Role of ovarian progesterone and potential role of prostaglandin F2alpha and prostaglandin E2 in modulating the uterine response to infectious bacteria in postpartum ewes. J Anim Sci. 2003;81(1):285‐293. 12597400 10.2527/2003.811285x

[8] Rietschel ET , Kirikae T , Schade FU , et al. Bacterial endotoxin: molecular relationships of structure to activity and function. FASEB J. 1994;8(2):217‐225. 8119492 10.1096/fasebj.8.2.8119492

[9] Lee JW , Paape MJ , Elsasser TH , Zhao X . Elevated milk soluble CD14 in bovine mammary glands challenged with *Escherichia coli* lipopolysaccharide. J Dairy Sci. 2003;86(7):2382‐2389. 12906056 10.3168/jds.S0022-0302(03)73832-6

[10] Akira S , Uematsu S , Takeuchi O . Pathogen recognition and innate immunity. Cell. 2006;124(4):783‐801. 16497588 10.1016/j.cell.2006.02.015

[11] Shan H , Lilly ST , Fischer DP , et al. Bacterial lipopolysaccharide induces an endocrine switch from prostaglandin F2alpha to prostaglandin E2 in bovine endometrium. Endocrinology. 2009;150(4):1912‐1920. 19056817 10.1210/en.2008-1379 PMC2706387

[12] Herath S , Fischer D , Werling D , et al. Expression and function of toll‐like receptor 4 in the endometrial cells of the uterus. Endocrinology. 2006;147(1):562‐570. 16223858 10.1210/en.2005-1113 PMC2738982

[13] Sweet MJ , Hume DA . Endotoxin signal transduction in macrophages. J Leukoc Biol. 1996;60(1):8‐26. 8699127 10.1002/jlb.60.1.8

[14] Guha M , Mackman N . LPS induction of gene expression in human monocytes. Cell Signal. 2001;13(2):85‐94. 11257452 10.1016/s0898-6568(00)00149-2

[15] Cronin JG , Turner ML , Goetze L , Bryant CE , Sheldon IM . Toll‐like receptor 4 and MYD88‐dependent signaling mechanisms of the innate immune system are essential for the response to lipopolysaccharide by epithelial and stromal cells of the bovine endometrium. Biol Reprod. 2012;86(2):51. 22053092 10.1095/biolreprod.111.092718 PMC4396703

[16] Fu Y , Bo L , Feng X , et al. Lipopolysaccharide increases toll‐like receptor 4 and downstream toll‐like receptor signaling molecules expression in bovine endometrial epithelial cells. Vet Immunol Immunopathol. 2013;151(1–2):20‐27. 23218938 10.1016/j.vetimm.2012.09.039

[17] Hoffmann PR , Berry MJ . The influence of selenium on immune responses. Mol Nutr Food Res. 2010;52(11):1273‐1280. 10.1002/mnfr.200700330 PMC3723386 18384097

[18] Duntas LH . Selenium and inflammation: underlying anti‐inflammatory mechanisms. Horm Metab Res. 2009;41(6):443‐447. 19418416 10.1055/s-0029-1220724

[19] Maehira F , Miyagi I , Eguchi Y . Selenium regulates transcription factor NF‐kappaB activation during the acute phase reaction. Clin Chim Acta. 2003;334(1–2):163‐171. 12867288 10.1016/s0009-8981(03)00223-7

[20] Hawkes WC , Kelley DS , Taylor PC . The effects of dietary selenium on the immune system in healthy men. Biol Trace Elem Res. 2001;81(3):189‐213. 11575678 10.1385/BTER:81:3:189

[21] Mihm S , Galter D , Dröge W . Modulation of transcription factor NF kappa B activity by intracellular glutathione levels and by variations of the extracellular cysteine supply. FASEB J. 1995;9(2):246‐252. 7781927 10.1096/fasebj.9.2.7781927

[22] Zhang Y , Xu Y , Chen B , Zhao B , Gao XJ . Selenium deficiency promotes oxidative stress‐induced mastitis via activating the NF‐κB and MAPK pathways in dairy cow. Biol Trace Elem Res. 2022;200(6):2716‐2726. 34455543 10.1007/s12011-021-02882-0

[23] Zhirong Z , Qiaojian Z , Chunjing X , et al. Methionine selenium antagonizes LPS‐induced necroptosis in the chicken liver via the miR‐155/TRAF3/MAPK axis. J Cell Physiol. 2021;236(5):4024‐4035. 33151563 10.1002/jcp.30145

[24] Zamamiri‐Davis F , Lu Y , Thompson JT , et al. Nuclear factor‐kappaB mediates over‐expression of cyclooxygenase‐2 during activation of RAW 264.7 macrophages in selenium deficiency. Free Radic Biol Med. 2002;32(9):890‐897. 11978490 10.1016/s0891-5849(02)00775-x

[25] Pathak R , Prasad S , Kumaresan A , Kaur M , Manimaran A , Dang AK . Alterations in cortisol concentrations and expression of certain genes associated with neutrophil functions in cows developing retention of fetal membranes. Vet Immunol Immunopathol. 2015;168(3–4):164‐168. 26384698 10.1016/j.vetimm.2015.09.003

[26] Werb Z . Biochemical actions of glucocorticoids on macrophages in culture. Specific inhibition of elastase, collagenase, and plasminogen activator secretion and effects on other metabolic functions. J Exp Med. 1978;147(6):1695‐1712. 210248 10.1084/jem.147.6.1695 PMC2184323

[27] Hillier SG , Tetsuka M . An anti‐inflammatory role for glucocorticoids in the ovaries. J Reprod Immunol. 1998;39(1–2):21‐27. 9786451 10.1016/s0165-0378(98)00011-4

[28] Roth JA , Kaeberle ML . Effect of glucocorticoids on the bovine immune system. J Am Vet Med Assoc. 1982;180(8):894‐901. 6177675

[29] Dong J , Qu Y , Li J , et al. Cortisol inhibits NF‐κB and MAPK pathways in LPS activated bovine endometrial epithelial cells. Int Immunopharmacol. 2018;56:71‐77. 29367089 10.1016/j.intimp.2018.01.021

[30] Cui L , Wang H , Lin J , et al. Progesterone inhibits inflammatory response in *E.coli*‐ or LPS‐stimulated bovine endometrial epithelial cells by NF‐κB and MAPK pathways. Dev Comp Immunol. 2020;105:103568. 31821816 10.1016/j.dci.2019.103568

[31] Bi CL , Wang H , Wang YJ , et al. Selenium inhibits Staphylococcus aureus‐induced inflammation by suppressing the activation of the NF‐κB and MAPK signalling pathways in RAW264.7 macrophages. Eur J Pharmacol. 2016;780:159‐165. 27036486 10.1016/j.ejphar.2016.03.044

[32] Livak KJ , Schmittgen TD . Analysis of relative gene expression data using real‐time quantitative PCR and the 2(‐Delta Delta C(T)) method. Methods. 2001;25(4):402‐408. 11846609 10.1006/meth.2001.1262

[33] Nikaido H . Molecular basis of bacterial outer membrane permeability revisited. Microbiol Mol Biol Rev. 2003;67(4):593‐656. 14665678 10.1128/MMBR.67.4.593-656.2003 PMC309051

[34] Raetz CR , Whitfield C . Lipopolysaccharide endotoxins. Annu Rev Biochem. 2002;71:635‐700. 12045108 10.1146/annurev.biochem.71.110601.135414 PMC2569852

[35] Hoshino K , Takeuchi O , Kawai T , et al. Cutting edge: toll‐like receptor 4(TLR4)‐deficient mice are hyporesponsive to lipopolysaccharide: evidence for TLR4 as the LPS gene product. J Immunol. 1999;162(7):749‐3752. 10201887

[36] Zhou M , Xu W , Wang J , et al. Boosting mTOR‐dependent autophagy via upstream TLR4‐MyD88‐MAPK signalling and downstream NF‐κB pathway quenches intestinal inflammation and oxidative stress injury. EBioMedicine. 2018;35:345‐360. 30170968 10.1016/j.ebiom.2018.08.035 PMC6161481

[37] Papp LV , Jun LU , Holmgren A , et al. From selenium to selenoproteins: synthesis, identity, and their role in human health. Antioxid Redox Signal. 2007;9(7):775‐806. 17508906 10.1089/ars.2007.1528

[38] Brayman M . The importance of selenium to human health. Lancet. 2000;356(9225):233‐241. 10963212 10.1016/S0140-6736(00)02490-9

[39] Minich WB . Selenium metabolism and biosynthesis of Selenoproteins in the human body. Biochemistry (Mosc). 2022;87(Suppl 1):S168‐S177. 35501994 10.1134/S0006297922140139 PMC8802287

[40] Fairweather‐Tait SJ , Bao Y , Broadley MR , et al. Selenium in human health and disease. Antioxid Redox Signal. 2011;14(7):1337‐1383. 20812787 10.1089/ars.2010.3275

[41] Vunta H , Belda BJ , Arner RJ , Channa Reddy C , vanden Heuvel JP , Sandeep Prabhu K . Selenium attenuates pro‐inflammatory gene expression in macrophages. Mol Nutr Food Res. 2008;52(11):1316‐1323. 18481333 10.1002/mnfr.200700346

[42] Fan Z , Wei Y , Hargrove JL , et al. Inhibition of TNF‐alpha induced ICAM‐1, VCAM‐1 and E‐selectin expression by selenium. Atherosclerosis. 2002;161(2):381‐386. 11888521 10.1016/s0021-9150(01)00672-4

[43] Ala M , Jafari RM , Nematian H , et al. Sodium selenite modulates IDO1/kynurenine, TLR4, NF‐κB and Bcl2/Bax pathway and mitigates acetic ccid‐induced colitis in rat. Cell Physiol Biochem. 2022;56(S1):24‐35. 35263537 10.33594/000000504

[44] Fisher AD , Verkerk GA , Morrow CJ , Matthews LR . The effects of feed restriction and lying deprivation on pituitary–adrenal axis regulation in lactating cows. Livest Prod Sci. 2002;73(2–3):255‐263.

[45] Nakao T , Grunert E . Effects of dystocia on postpartum adrenocortical function in dairy cows. J Dairy Sci. 1990;73(10):2801‐2806. 2178172 10.3168/jds.S0022-0302(90)78967-9

[46] Weiss WP , Todhunter DA , Hogan JS , Smith KL . Effect of duration of supplementation of selenium and vitamin E on periparturient dairy cows. J Dairy Sci. 1990;73(11):3187‐3194. 2273147 10.3168/jds.S0022-0302(90)79009-1

[47] Salman S , Dinse D , Khol‐Parisini A , et al. Colostrum and milk selenium, antioxidative capacity and immune status of dairy cows fed sodium selenite or selenium yeast. Arch Anim Nutr. 2013;67(1):48‐61. 23298256 10.1080/1745039X.2012.755327

[48] Dimri U , Ranjan R , Sharma MC , Varshney VP . Effect of vitamin E and selenium supplementation on oxidative stress indices and cortisol level in blood in water buffaloes during pregnancy and early postpartum period. Tropl Anim Health Prod. 2010;42(3):405‐410. 10.1007/s11250-009-9434-4 19763870

[49] Morey M , Serras F , Baguñà J , Hafen E , Corominas M . Modulation of the Ras/MAPK signalling pathway by the redox function of selenoproteins in Drosophila melanogaster. Develop Biol. 2001;238(1):145‐156. 11784000 10.1006/dbio.2001.0389

[50] Huang JQ , Ren FZ , Jiang YY , Xiao C , Lei XG . Selenoproteins protect against avian nutritional muscular dystrophy by metabolizing peroxides and regulating redox/apoptotic signaling. Free Radic Biol Med. 2015;83:129‐138. 25668720 10.1016/j.freeradbiomed.2015.01.033

[51] Kretzremy C , Arrigo AP . Selenium: a key element that controls NF‐kappa B activation and I kappa B alpha half life. Biofactors. 2001;14(1–4):117‐125. 11568448 10.1002/biof.5520140116

[52] Raha S , Yumnam S , Hong GE , et al. Naringin induces autophagy‐mediated growth inhibition by downregulating the PI3K/Akt/mTOR cascade via activation of MAPK pathways in AGS cancer cells. Int J Oncol. 2015;47(3):1061‐1069. 26201693 10.3892/ijo.2015.3095

[53] Papp LV , Holmgren A , Khanna KK . Selenium and selenoproteins in health and disease. Antioxid Redox Signal. 2010;12(7):793‐795. 19905883 10.1089/ars.2009.2973

[54] Reuter S , Gupta SC , Chaturvedi MM , Aggarwal BB . Oxidative stress, inflammation, and cancer: how are they linked. Free Radic Biol Med. 2010;49(11):1603‐1616. 20840865 10.1016/j.freeradbiomed.2010.09.006 PMC2990475

