# Supplementary material for: Selenium suppressed the LPS‐induced inflammation of bovine endometrial epithelial cells through NF‐κB and MAPK pathways under high cortisol background
Source: J Cell Mol Med. 2023 Apr 11;27(10):1373–83. doi: 10.1111/jcmm.17738 (PMC10183709; doi:10.1111/jcmm.17738)
Supplement: Supplementary file 1 — Supinfo [file JCMM-27-1373-s001.zip › JCMM_17738_Suppl file1-revised.docx]

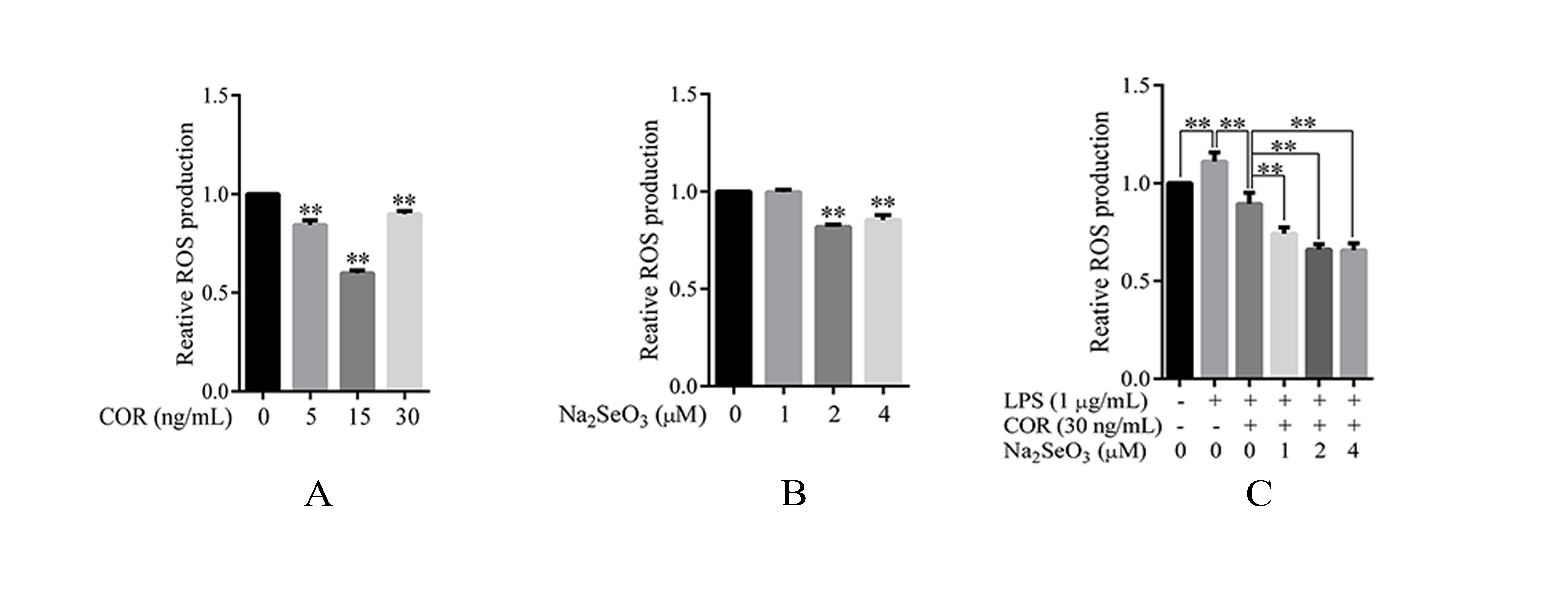


The effect of cortisol (COR) and Na_2_SeO_3_ on the level of reactive oxygen species (ROS) in primary bovine endometrial epithelial cells. The cells were treated with COR (A) or Na_2_SeO_3_ (B) for 12 h. (C) The cells were pretreated with Na_2_SeO_3_ for 12 h, and were then treated with COR and lipopolysaccharide (LPS) for an additional 12 h. The data were presented as the means ± SEM (n = 3). * *p* < 0 .05, ** *p* < 0.01.
