# Supplementary figures and images for: Selenium suppressed the LPS‐induced inflammation of bovine endometrial epithelial cells through NF‐κB and MAPK pathways under high cortisol background
Source: J Cell Mol Med. 2023 Apr 11;27(10):1373–83. doi: 10.1111/jcmm.17738 (PMC10183709; doi:10.1111/jcmm.17738)

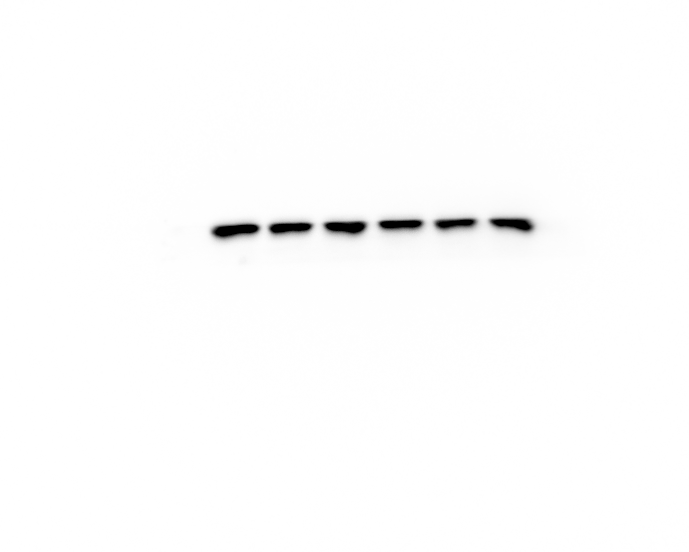

Supplement: Supplementary file 1 — Supinfo [file JCMM-27-1373-s001.zip › JCMM_17738_GAPDH FOR P65-FIG6.tif]

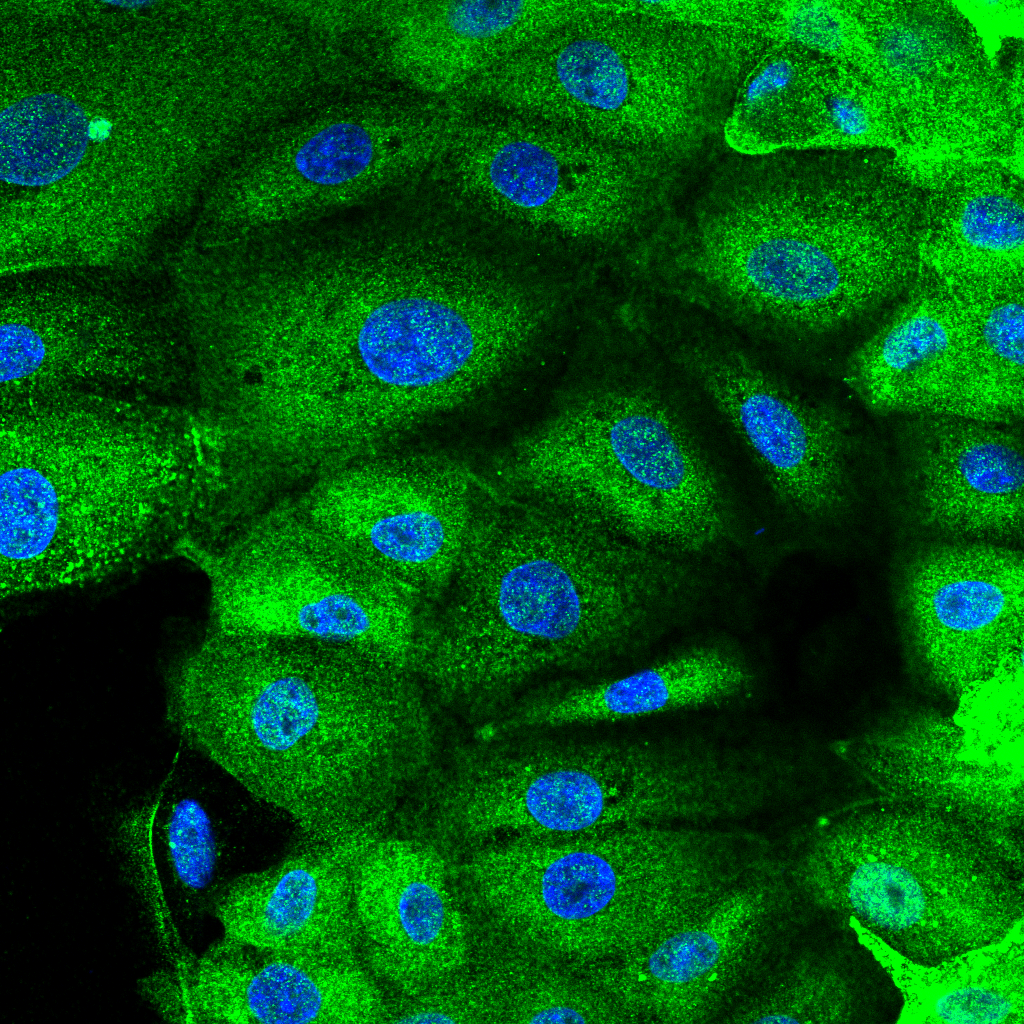

Supplement: Supplementary file 1 — Supinfo [file JCMM-27-1373-s001.zip › JCMM_17738_LPS.tif]
